# A Rare Case of Left Internal Mammary Artery Transection During Pericardiocentesis

**DOI:** 10.1016/j.jaccas.2025.104041

**Published:** 2025-06-25

**Authors:** Shaun Abid, Anton Stolear, Lila Kaminsky, Samdish Sethi, David Narotsky, Chirag Shah, Matthew Seigerman

**Affiliations:** a Department of Internal Medicine, Yale New Haven Health, Bridgeport Hospital, Bridgeport, Connecticut, USA; b Division of Cardiology, Department of Internal Medicine, Yale New Haven Health, Bridgeport Hospital, Bridgeport, Connecticut, USA; c Division of Cardiology, Interventional and Structural Heart Disease, Yale New Haven Health, Bridgeport Hospital, Bridgeport, Connecticut, USA

**Keywords:** anticoagulation management, complication, echocardiography, pericardial effusion, tamponade, vascular embolization

## Abstract

**Background:**

Pericardiocentesis is a common procedure for managing pericardial effusions, but it carries risks for vascular injury, particularly in patients with coagulopathies.

**Case Summary:**

A 72-year-old man with factor V Leiden mutation and sick sinus syndrome (status post pacemaker placement), presented with dizziness, dyspnea, and chest pain. Echocardiography revealed a large pericardial effusion with tamponade physiology, necessitating pericardiocentesis. After drain removal, the patient developed hypotension and bradycardia. Imaging identified active bleeding from a distal branch of the left internal mammary artery (LIMA). Hemostasis was achieved via embolization and microcoil placement. The patient recovered with no reaccumulation of effusion or hematoma.

**Discussion:**

This rare case of LIMA injury following pericardiocentesis highlights an underrecognized complication, particularly in patients with coagulation disorders. It underscores the need for vigilant postprocedural monitoring and the role of advanced imaging in diagnosing vascular injuries.

**Take-Home Message:**

LIMA injury is a rare but serious complication of pericardiocentesis, requiring prompt-recognition and intervention.

## Graphical Abstract

**Figure:**
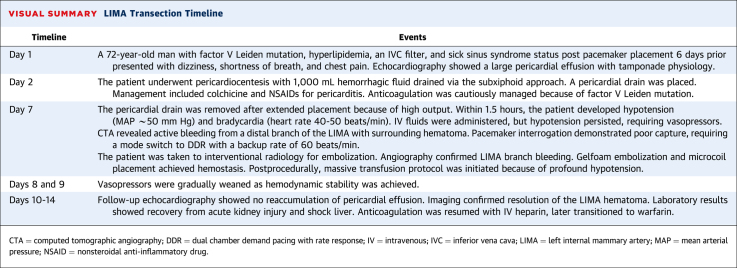

**Take-Home Messages**

• Awareness of rare vascular injuries, such as LIMA damage, is crucial in patients with complex histories undergoing invasive procedures.
• In patients with factor V Leiden mutation, pericardiocentesis must be performed with careful preprocedural coagulation assessment, cautious anticoagulation adjustments, and close postprocedural monitoring for signs of bleeding or thrombotic complications.

## History of Presentation

A 72-year-old man underwent a dual-chamber pacemaker procedure 6 days prior to presentation and subsequently developed progressively worsening dizziness, shortness of breath, and chest pain, ultimately prompting him to seek medical care. On physical examination, the cardiovascular system showed a regular rate and rhythm and normal S1 and S2, with no murmurs or gallops. There was no jugular venous distension, and the point of maximal impulse was nondisplaced. Pulses were normal, and no friction rub was detected. Pulmonary effort was normal without respiratory distress, and breath sounds were clear, with no stridor, wheezing, rhonchi, or rales. The chest wall was nontender. The abdominal examination revealed a flat, soft, nontender abdomen with normal bowel sounds, no distension, and no masses. There was no edema in the lower extremities. However, motor weakness was noted.

## Past Medical History

The patient's medical history included hyperlipidemia, factor V Leiden mutation requiring long-term anticoagulation with warfarin, inferior vena cava filter placement, and sick sinus syndrome managed with dual-chamber pacemaker placement 6 days prior.

## Differential Diagnosis

The differential diagnosis included pacemaker lead perforation, pericardial effusion with tamponade, pacemaker malfunction, myocardial infarction, heart failure, pulmonary embolism, and coagulopathy-related complications from factor V Leiden mutation and anticoagulation therapy.

## Investigations

On presentation, the patient's vital signs were notable for blood pressure of 116/80 mm Hg, a pulse of 63 beats/min, a temperature of 97.4 °F (36.3 °C) orally, a respiratory rate of 18 breaths/min, and oxygen saturation of 98% on room air. Laboratory findings were significant for a hemoglobin level of 12 g/dL (baseline 14 g/dL; reference range: 13.2-17.1 g/dL) and a troponin level of 9 ng/L (reference range: <12 ng/L). Echocardiography revealed a large pericardial effusion with tamponade physiology (Video 1) and a plethoric inferior vena cava with reduced respiratory variability (Video 2).

## Management

The patient underwent pericardiocentesis, during which 1,000 mL hemorrhagic fluid was successfully drained via the subxiphoid approach under fluoroscopic guidance. After entry into the pericardial space, position was confirmed by successful aspiration of blood, followed by fluoroscopic visualization of the wire looping around the heart within the pericardial space before advancing the sheath. Intrapericardial pressures were recorded, with a pretap pressure of 20 mm Hg and a post-tap pressure of 0 mm Hg. A bubble study was not performed. A pericardial drain was placed, and colchicine and nonsteroidal anti-inflammatory drugs were initiated to manage pericarditis. Postoperative chest radiography showed a pericardial drain in place, with left chest pacer leads terminating in the right atrial appendage and right ventricle. The cardiac silhouette was enlarged, and no pneumothorax was present. Careful anticoagulation management was necessary because of the patient's factor V Leiden mutation. The pericardial drain remained in place longer than expected because of continued high output, likely related to the factor V Leiden mutation, and was removed on the morning of the sixth day.

**Figure 1:**
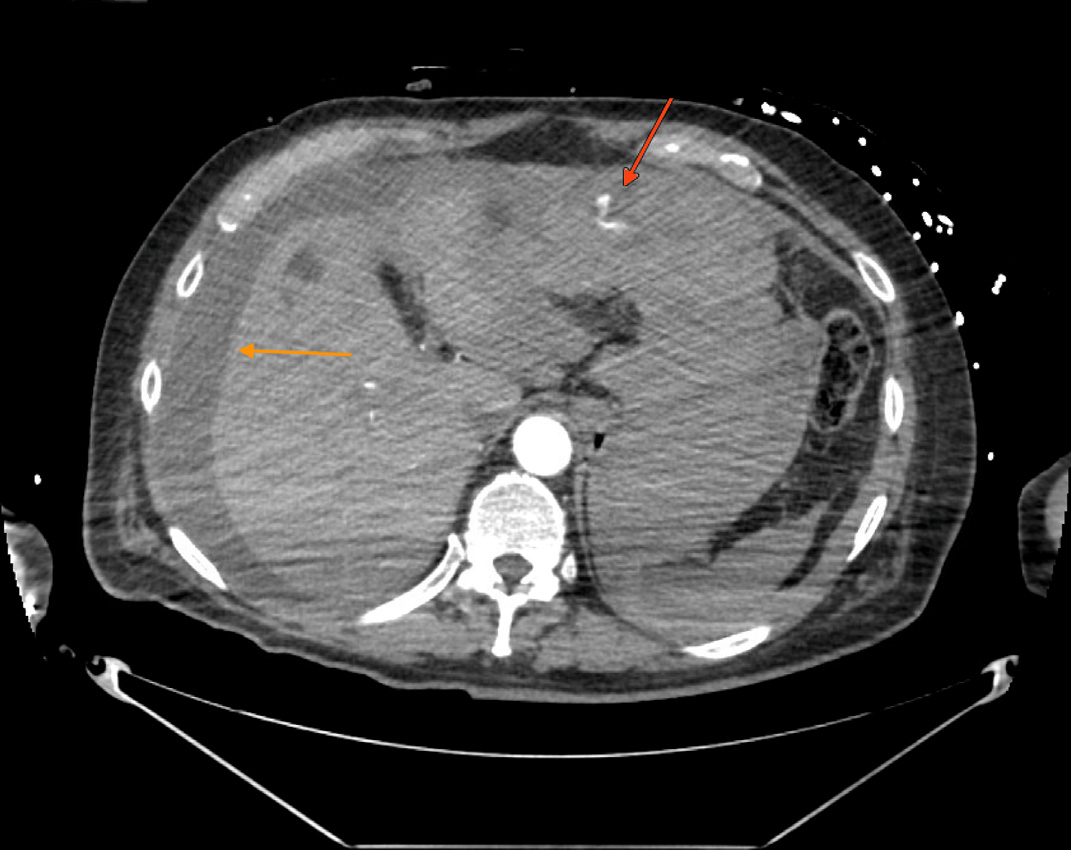
Computed Tomographic Angiography of the Abdomen and Pelvis With Left Internal Mammary Artery Bleed Axial view depicting active arterial contrast extravasation and bleed in the epigastric region of the peritoneum (red arrow), surrounded by hemoperitoneum (orange arrow).

**Figure 2:**
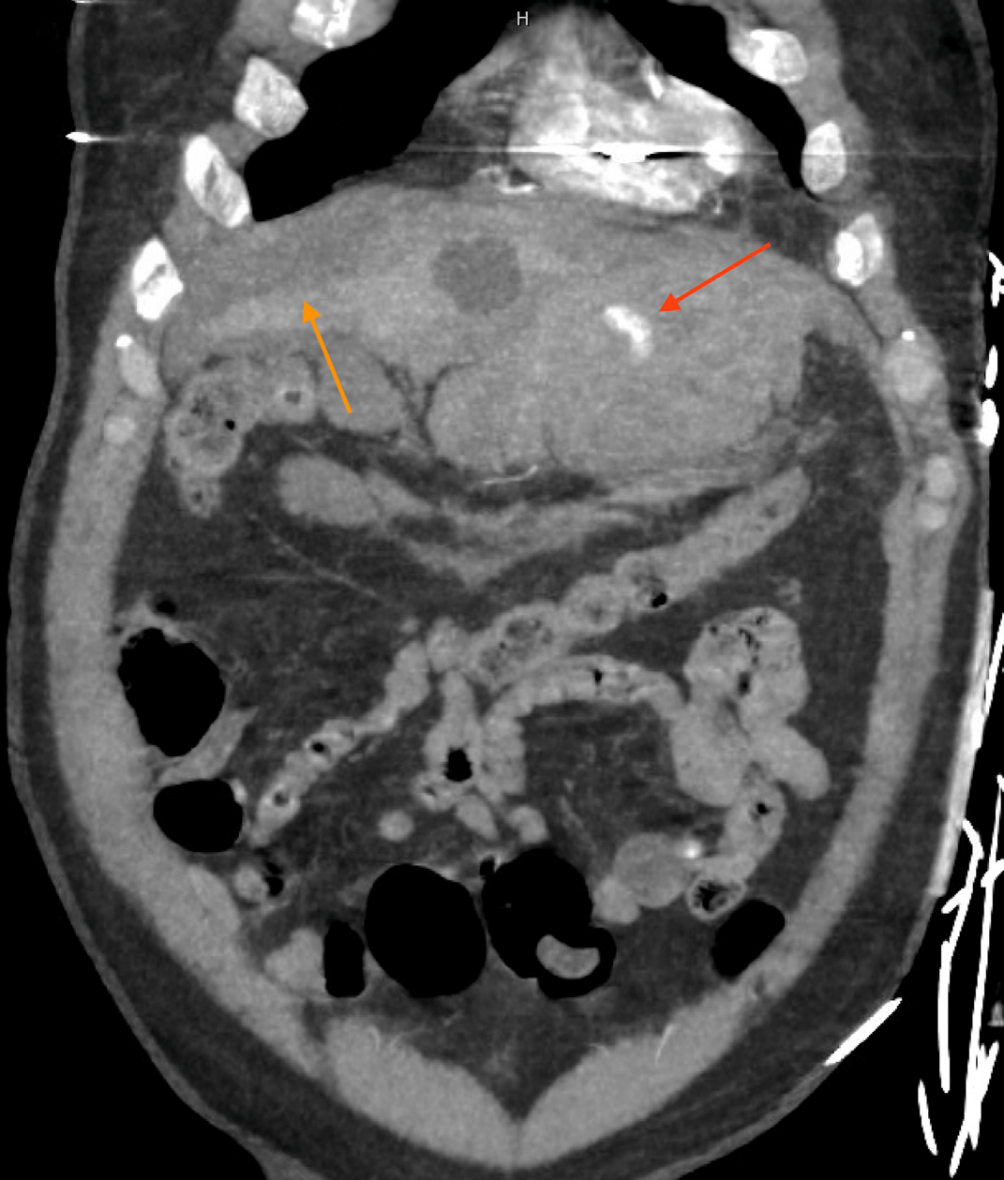
Computed Tomographic Angiography of the Abdomen and Pelvis With Left Internal Mammary Artery Bleed Coronal view depicting active arterial contrast extravasation and bleed in the epigastric region of the peritoneum (red arrow), surrounded by hemoperitoneum (orange arrow).

Within 1.5 hours of drain removal, the patient developed hypotension (mean arterial pressure ∼50 mm Hg) and bradycardia (heart rate 40-50 beats/min). Despite 3 L of intravenous fluid boluses, hypotension persisted, requiring initiation of 3 vasopressors (norepinephrine, phenylephrine, and vasopressin) to support hemodynamic status. Serial bedside echocardiography showed no reaccumulation of pericardial effusion or evidence of right heart strain, while chest radiography revealed stable bibasilar atelectasis without pneumothorax. Computed tomographic angiography of the chest, abdomen, and pelvis identified active arterial bleeding from a distal branch of the left internal mammary artery (LIMA), with surrounding hemoperitoneum (Figures 1 and 2). Pacemaker interrogation demonstrated poor capture, prompting a mode adjustment to dual-chamber demand pacing with rate response, with a backup rate of 60 beats/min.

**Figure 3:**
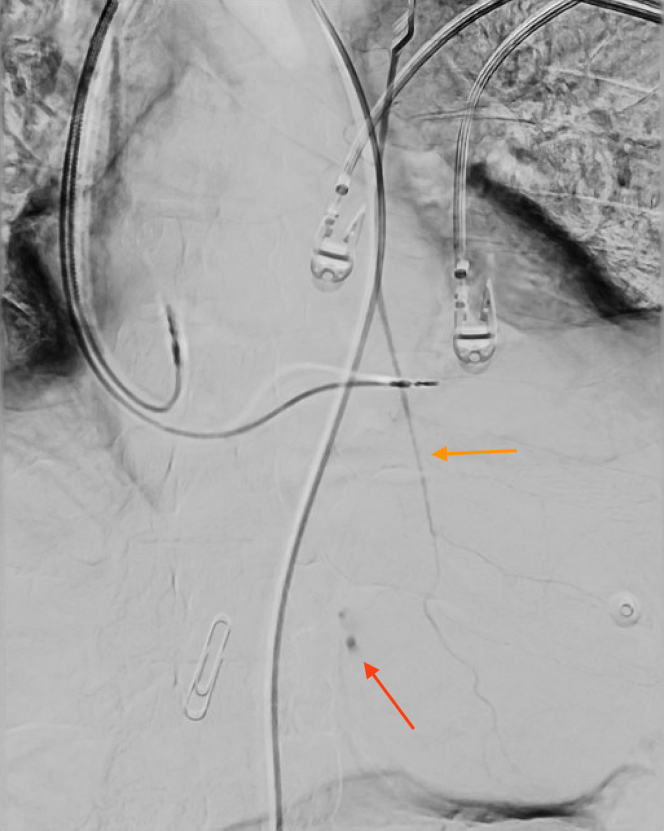
Fluoroscopic View of the Left Internal Mammary Artery Active extravasation from a small distal branch of the left internal mammary artery (LIMA) (red arrow). LIMA descending into epigastric region (orange arrow).

**Figure 4:**
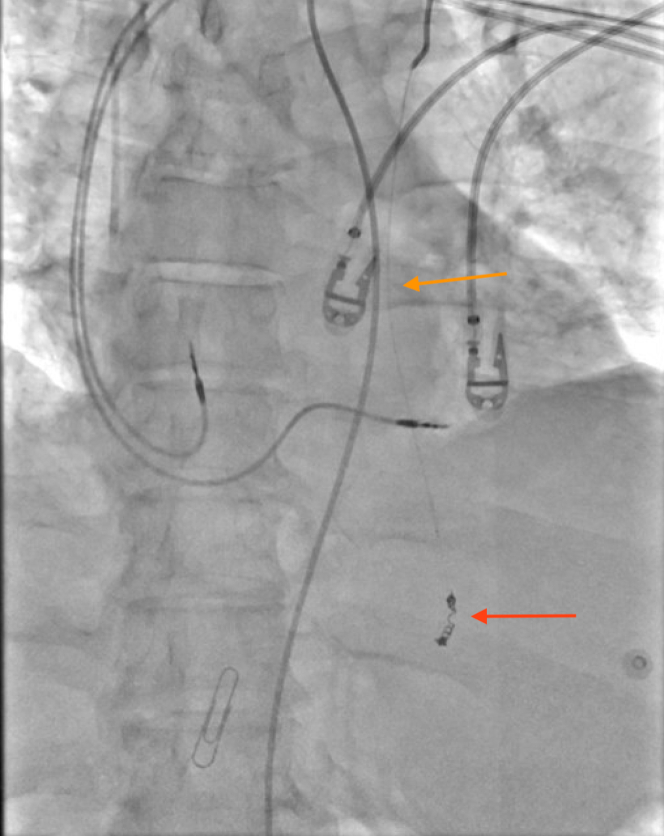
Fluoroscopic View of Coil Microcoil after placement in the distal left internal mammary artery (LIMA) (red arrow). LIMA with wire descending toward coil (orange arrow).

The patient was emergently taken to interventional radiology, where angiography confirmed active extravasation from a distal branch of the LIMA (Figure 3). Hemostasis was successfully achieved using Gelfoam (Pfizer) embolization followed by microcoil placement (Figure 4), with confirmation on repeat angiography. Following the procedure, the patient experienced significant clinical deterioration, presenting with profound hypotension that was unresponsive to initial resuscitation efforts. An emergent Cordis catheter was placed to facilitate the massive transfusion protocol, which included the administration of 5 U packed red blood cells, 3 U fresh frozen plasma, and 1 U platelets. The deterioration following the procedure was likely not attributable to continued bleeding, as hemostasis had been confirmed angiographically. Instead, it was more plausibly related to delayed stabilization of the patient's hemodynamic status, a consequence of the significant intravascular depletion and physiological stress sustained prior to intervention. Over the subsequent 12 hours, the patient's condition improved as vasopressors were gradually weaned, and hemodynamic stability was restored.

## Outcome and Follow-Up

Over the subsequent days, the patient's condition improved with careful monitoring. Follow-up echocardiography showed no reaccumulation of the pericardial effusion, and serial imaging confirmed resolution of the hematoma. Laboratory parameters, including renal function and liver enzymes, improved, indicating recovery from acute kidney injury and shock liver. Anticoagulation was cautiously resumed with intravenous heparin under high-risk bleeding protocols, and the patient was transitioned back to warfarin once stable. Follow-up 1 month later revealed that the patient was actively participating in cardiac rehabilitation and gradually restoring physical activity to baseline levels.

## Discussion

Blind pericardiocentesis, often performed in emergencies, relies on anatomical landmarks and carries a higher risk for complications. Studies have identified the subxiphoid approach, targeting Larrey's triangle at a 30° angle toward the left midclavicular line, as the safest and most effective blind technique.^1^ This approach follows an extrapleural route, reducing the likelihood of injury to structures such as the coronary arteries, pericardial arteries, and internal mammary arteries.^2^ In contrast, echocardiography-guided pericardiocentesis provides superior safety and precision. By using contrast-enhanced 2-dimensional echocardiography, clinicians can pinpoint the optimal needle insertion site, avoid vital structures, and confirm fluid removal. This minimally invasive technique has become the gold standard, particularly for critically ill patients, significantly lowering risks and improving outcomes.^3^

The most severe complications of pericardiocentesis include laceration or perforation of the myocardium or coronary vessels. Other risks include air embolism, pneumothorax, arrhythmias, and accidental puncture of the peritoneal cavity or abdominal organs.^4^ Laceration of the LIMA is an exceedingly rare complication, with only 2 prior instances documented in PubMed and Google searches, along with 1 case of a LIMA pseudoaneurysm induced by pericardiocentesis.^5^, ^6^, ^7^

This case demonstrates a rare but severe complication of pericardiocentesis. The LIMA originates from the subclavian artery and runs along the inner thoracic wall, approximately 2 to 3 cm lateral to the sternum, bifurcating at the sixth or seventh intercostal space into the musculophrenic and superior epigastric arteries. These branches supply the diaphragm, pericardium, and abdominal wall. During the subxiphoid approach, it is likely that the needle transected the LIMA, resulting in arterial injury. The drain likely tamponaded the vessel while in place, delaying the recognition of this complication.

Although LIMA injury is more commonly associated with blunt or penetrating chest trauma,^8^ it is rarely reported as a complication of pericardiocentesis. In this case, the patient's pacemaker related effusion, factor V Leiden mutation, and procedural complexities created a unique clinical challenge. This underscores the importance of advanced imaging and a multidisciplinary approach in recognizing and managing such rare complications.

Patients with factor V Leiden mutation require meticulous intraprocedural and periprocedural management to balance the risks for thrombosis and bleeding. Preprocedurally, it is essential to assess the patient's coagulation status, including international normalized ratio, activated partial thromboplastin time, and platelet count, particularly in those on long-term anticoagulation. A thorough preprocedural imaging evaluation is crucial to identify any aberrant vasculature and select the safest approach. During the procedure, multiple modalities should be used to confirm access to the pericardial space before advancing the wire and sheath and to verify proper placement of the pericardial drain. Postprocedurally, these patients must be monitored vigilantly for bleeding complications, given their increased susceptibility because of prior anticoagulation use and potential procedural vascular injury. Drain output should be frequently assessed for an increase in bloody discharge, which may indicate ongoing hemorrhage or delayed vessel injury. Serial hemoglobin and hematocrit measurements, along with coagulation panels, should be checked to detect early signs of bleeding. In cases of continued high pericardial drain output, further imaging such as echocardiography or computed tomographic angiography may be required to evaluate for persistent pericardial hemorrhage or new vascular injury. The decision to restart anticoagulation postprocedure should be individualized, carefully balancing the risks for thrombosis and rebleeding. A multidisciplinary team involving cardiology, hematology, and critical care teams ensures optimal management in these high-risk patients, reducing both thrombotic and hemorrhagic complications.

## Conclusions

LIMA injury is a rare but significant complication of pericardiocentesis, particularly in patients with coagulopathy. Vigilant postprocedural monitoring and the use of advanced imaging are essential for timely recognition and effective management of vascular injuries.

**Visual Summary:** LIMA Transection Timeline

Timeline	Events
Day 1	A 72-year-old man with factor V Leiden mutation, hyperlipidemia, an IVC filter, and sick sinus syndrome status post pacemaker placement 6 days prior presented with dizziness, shortness of breath, and chest pain. Echocardiography showed a large pericardial effusion with tamponade physiology.
Day 2	The patient underwent pericardiocentesis with 1,000 mL hemorrhagic fluid drained via the subxiphoid approach. A pericardial drain was placed. Management included colchicine and NSAIDs for pericarditis. Anticoagulation was cautiously managed because of factor V Leiden mutation.
Day 7	The pericardial drain was removed after extended placement because of high output. Within 1.5 hours, the patient developed hypotension (MAP ∼50 mm Hg) and bradycardia (heart rate 40-50 beats/min). IV fluids were administered, but hypotension persisted, requiring vasopressors. CTA revealed active bleeding from a distal branch of the LIMA with surrounding hematoma. Pacemaker interrogation demonstrated poor capture, requiring a mode switch to DDR with a backup rate of 60 beats/min. The patient was taken to interventional radiology for embolization. Angiography confirmed LIMA branch bleeding. Gelfoam embolization and microcoil placement achieved hemostasis. Postprocedurally, massive transfusion protocol was initiated because of profound hypotension.
Days 8 and 9	Vasopressors were gradually weaned as hemodynamic stability was achieved.
Days 10-14	Follow-up echocardiography showed no reaccumulation of pericardial effusion. Imaging confirmed resolution of the LIMA hematoma. Laboratory results showed recovery from acute kidney injury and shock liver. Anticoagulation was resumed with IV heparin, later transitioned to warfarin.

CTA = computed tomographic angiography; DDR = dual chamber demand pacing with rate response; IV = intravenous; IVC = inferior vena cava; LIMA = left internal mammary artery; MAP = mean arterial pressure; NSAID = nonsteroidal anti-inflammatory drug.

## Funding Support and Author Disclosures

The authors have reported that they have no relationships relevant to the contents of this paper to disclose.

## Appendix

Video 1 Echocardiography with tamponade: subcostal view depicting a large pericardial effusion, causing right ventricular collapse on diastole.

Video 2 Echocardiogram of inferior vena cava: subcostal view depicting a plethoric inferior vena cava. Pacemaker leads can also be seen entering the atrium of the heart.
